# Service-learning Curriculum Design and Implementation at the University of Toronto Faculty of Medicine

**DOI:** 10.15694/mep.2019.000141.1

**Published:** 2019-06-21

**Authors:** Leedan Cohen, Fok-Han Leung, Chika Oriuwa, Roxanne Wright

**Affiliations:** 1 University of Toronto

**Keywords:** service-learning, community-engaged learning, pedagogy, undergraduate medical education, service-learning model

## Abstract

This article was migrated. The article was marked as recommended.

Community service-learning is an integral component of the undergraduate medical experience, as it provides students with the opportunity to respond to and address societal issues. Students at the University of Toronto, Faculty of Medicine have traditionally participated in a service-learning curriculum that required them to choose placement opportunities from a centrally- developed catalogue of options, with no continuity between the university and the community from year to year. The mandatory service-learning placement was re-designed under the advisement of long-standing community partners, community-engaged physicians, and academics. The new model centralizes the relationship between faculty tutors and community partners, who act as co-educators for the medical students, with tutors serving as the primary link to community organizations. The University of Toronto’s Faculty of Medicine is the first Canadian medical institution to implement this innovative curricular model.

## Background: Vision and objectives of new curriculum

Over the last 20 years, the Faculty of Medicine at the University of Toronto has included some form of community-situated learning in its undergraduate curriculum. Previous iterations have varied in their focus and intention. The current iteration is Health in Community (HC), a mandatory longitudinal two-year curriculum which focuses on the development and formation of physician response to changing community and societal issues and concerns. In first year, students explore concepts related to interprofessional teams, social determinants of health, and specific modalities of community care including home care and group homes. In second year, students participate in a service-learning placement for the entirety of the academic year, supported by several small-group tutorial sessions facilitated by a physician and an allied health practitioner. The model of service-learning developed for this experience is adapted from Tania D. Mitchell’s *critical service-learning* model, and aims to change systems and prioritize community-identified need ( Mitchell, 2008). The curriculum seeks to investigate the following questions:

- Why are some people healthier than others?
- What is community?
- Where is community?

The course objectives are also linked closely with the CanMEDS Roles and the Medical Council of Canada ‘Medical Expert’ objectives in Population Health. The CanMEDS Roles include: medical expert, communicator, collaborator, leader, health advocate, scholar and professional ( Royal College of Physicians and Surgeons of Canada, 2019).

**Figure 1.**
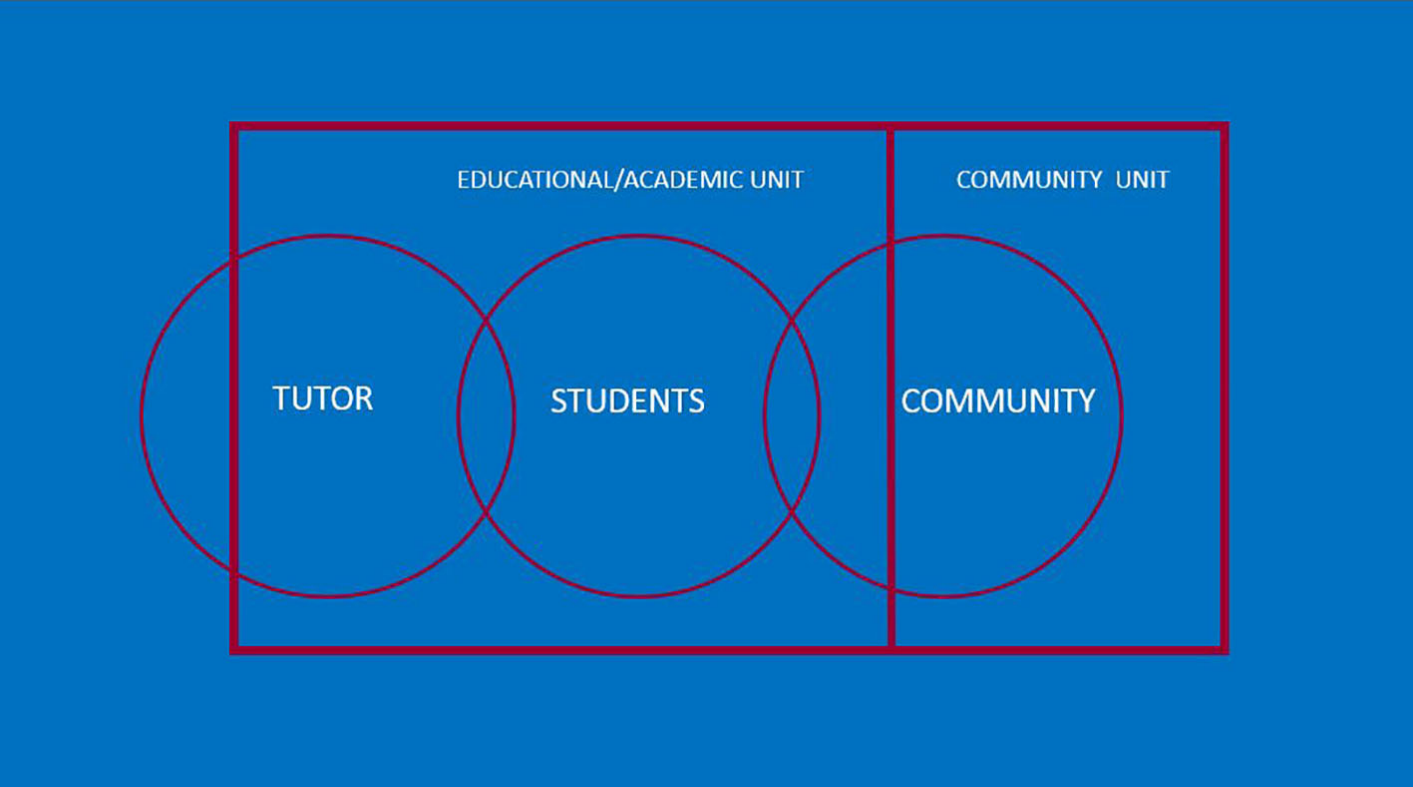
Previous model of community engagement

In the original model of service-learning used in the HC curriculum, students were arranged into small tutorial groups of 6-8, overseen by two tutors who are health care professionals (usually a family physician and an allied health practitioner). Each student was given the opportunity to select a community organization that they were interested in engaging with. Throughout the course of the year, the students were given various assignments and field work experiences. The tutorial group met about 5 times per year along with their tutors, to discuss their community placements and various experiences. In this model, the student was the link between the community organization and the tutors ( figure 1).

## New Curriculum: Consultation Process and Student Impact

In 2017, the Health in Community curriculum underwent a formal process of internal examination, with the intention of investigating the optimal way to engage students in community. Meetings and discussions with long- standing community partners, academics, community-engaged physicians and staff in community-engaged roles were arranged to gather important information on how the previous curriculum was running, whether it was impactful, and how it might be able to be improved. Broad questions were discussed, such as “what do you think medical students need to understand about working in community?” Key themes and outcomes from these meetings were collected. Two major themes emerged: a) community organizations benefited tremendously from having medical students as a part of their team, however, they expressed an interest in developing a deeper relationship with the university as a whole, and b) that impactful community engagement should acknowledge the role of the community partner as a co-educator and equal partner in the education of medical students around community involvement.

**Figure 2.**
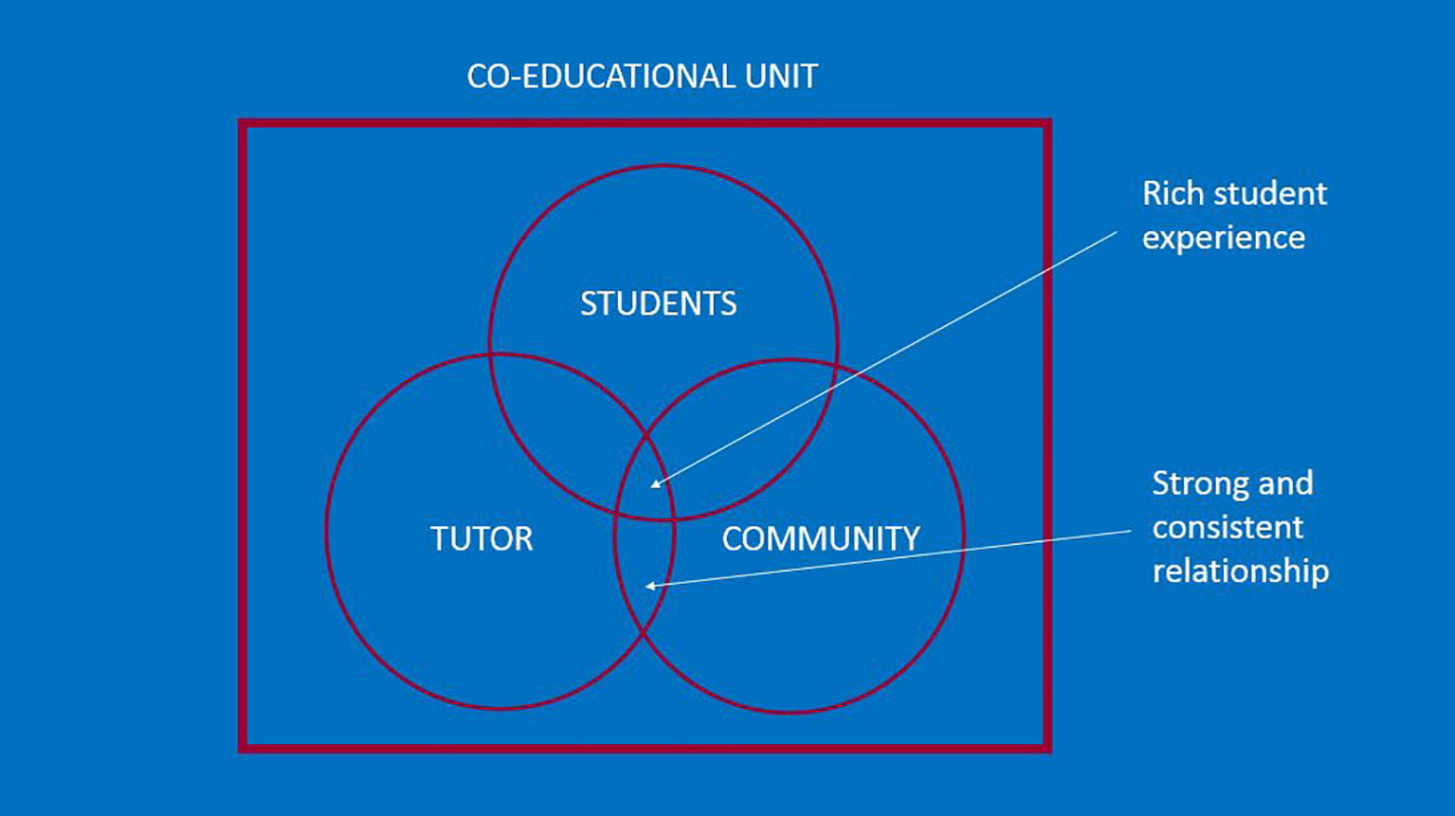
New model of community engagement

Upon review, the decision was made to re-design the structure of the Health in Community curriculum to integrate community partner needs and to further integrate service-learning into the curriculum. While the first year portion of the curriculum is largely unchanged, the second year portion which involves the service-learning component was adjusted. Tutors became the primary link between students and community organizations, by being permanently connected to the community partner organization. Unlike the previous model which saw students rotating in and out of a community placement year after year ( fig. 1), a tutor/faculty member is less ‘transient’ and better positioned to form a solid, consistent and supportive relationship with community partners. Thus, the community organizations are supported by the faculty tutors to help with medical student education and orientation. In this model, the tutorial group is assigned to a tutor and thereby, to a community organization, on an ongoing basis ( figure 2).

This new model allows for a richer student experience and a stronger and more consistent relationship between the medical community, the university and community partners. The goals of the curriculum include helping students develop community relationships that will be meaningful to future physicians and community partners, and to develop sustainable relationships with these partnerships over the years. Better relationships should lead to more meaningful opportunities for engagement in communities. A critical service-learning model, with a focus on social justice orientation, reorienting systems and building authentic relationships is emphasized ( Mitchell, 2008). This model also decreases various administrative burdens that were previously required in the former curriculum, where administrators were required to assist in matching student requests for community organizations as they applied to service-learning placements that were centrally developed. This novel model has been met with enthusiasm from community partners, educators and university administrators. This curriculum is the first of its kind in a Canadian medical school.

## Detailed Schedule

The Health in Community curriculum spans the first two years of medical training. In the first year, students will be oriented to the concept of service-learning and social determinants of health. At the end of the first year of training, community organizations are introduced to the students by their tutors.

At the start of the second year, tutorial groups and tutors visit their community partner site for a tutorial session which serves as an orientation. In subsequent field visits, students focus on learning about the community and community partner, paying specific attention to addressing challenges and discussing possible solutions. On these field experiences, students participate, observe, contribute and learn about advocacy. Field visits are punctuated with small-group tutorial sessions which offer opportunity for reflection as well as debate and discussion of major themes and topics connected to community-engaged learning. At the end of the year, students, community partners, faculty and administrators attend a Community Forum, where students prepare poster presentations to share their experiences with community partners, faculty and peers.

## Environmental Scan

When analyzing the current service-learning models at other medical education institutions across Canada, it became clear that the University of Toronto is providing a novel learning experience to its medical students. As an accreditation requirement, service-learning is offered in some form at every Canadian medical school. Flexibility in the delivery of this requirement, however, means that experiences vary greatly between institutions. The University of Toronto’s curriculum provides a particularly in-depth level of exposure with a mandatory service learning community placement for all students. With the tutor-community dyad forming a co-educational unit, the academic-community relationship is highlighted as the foundation of the work; this can mitigate real or perceived exploitation in either direction.

## Deliverables

Upon completion of the community partnership, each tutorial group participates in a conference-style poster presentation, attended by staff, faculty, peers and community partners. This allows the medical students to provide a critical reflection upon their journey with their partner organization.

In addition to making an investment of time in supporting the activities of their partner organization, each group of students needs to make a contribution to their organization by working to address a community-identified need through the creation of a resource (i.e., health education materials, programs or initiatives to enhance their work, quality improvement projects, etc.). This work is not assessed, and is developed with the community partner.

Lastly, there are also transformational outcomes for all stakeholders involved. Relationships are developed between first and second year students, students and community partners, faculty and community partners, and tutors and community partners.

## Conclusions

The community-based service-learning model at the University Of Toronto Faculty Of Medicine has been revised to place community at the head and heart of its mandate. The impetus for change was in response to requests from communities to develop longer and more genuine relationships. The direction of change was directed by community stakeholder consultation. The result is a model which is simple in concept, but rich in lessons for medical students and educators alike.

## Take Home Messages

• Service-learning has the potential to encourage students to create social change
• Community-based service-learning curriculum can be more effective when stakeholders are integrated with intention
• The model of service-learning used at the University of Toronto represents a method for integrating all educational stakeholders and providing continuity in relationships between campus and community

## Notes On Contributors

**Leedan Cohen** and **Chika Oriuwa** are third year medical students at the University of Toronto, Faculty of Medicine. They work together as student liaisons for the Health in Community course at the University, with the goals of improving service-learning and community relationships with medical professionals.

**Roxanne Wright** is the Experiential Learning Lead for the MD Program in the Faculty of Medicine at the University of Toronto.

**Dr. Fok-Han Leung** is the Director of the Health in Community curriculum for the MD Program in the Faculty of Medicine at the University of Toronto.
