# Supplementary material for: Service-learning Curriculum Design and Implementation at the University of Toronto Faculty of Medicine
Source: MedEdPublish (2016). 2019 Jun 21;8:141. [Version 1] doi: 10.15694/mep.2019.000141.1 (PMC10712482; doi:10.15694/mep.2019.000141.1)
Supplement: Peer review history

# Reviewer report: Reviewer response for version 1

**DOI:** 10.21956/mep.19857.r29983

**Published:** 2019-06-24

**Authors:** Hui Meng Er

**Affiliations:** 1 International Medical University

This review has been migrated. The reviewer awarded 4 stars out of 5 This is an interesting paper that describes a redesigned service-learning curriculum based on feedback from the community partners, academics, community-engaged physicians and staff in community-engaged roles. As students are the important stakeholders, was their feedback obtained and considered in the redesign? The new model with tutor as the primary link with the community aims to enhance the relationship with the community and enrich the student experience, as well as reduce the administrative burden in organising the community activities. Although this is at the early stage of implementation, evaluative findings of the new curriculum including achievement of learning outcomes as well as challenges in implementation (if any) should be reported. These will be useful to others who are considering to contextualise the model to their education environment.

Reviewer Expertise:

NA

No decision status is available

# Reviewer report: Reviewer response for version 1

**DOI:** 10.21956/mep.19857.r29982

**Published:** 2019-06-22

**Authors:** Tan Nguyen

**Affiliations:** 1 Deakin University

This review has been migrated. The reviewer awarded 5 stars out of 5 It is fantastic to see curriculum design that considers the needs of the community. Consumer involvement and co-design with consumers is absolutely necessary in assuring fitness to practice is promoted and maintained. It would be particularly beneficial to gain some insights by the medical students and the community partners regarding the process, the benefits and barriers to curriculum design.

Reviewer Expertise:

NA

No decision status is available
